# Doping practices in international weightlifting: analysis of sanctioned athletes/support personnel from 2008 to 2019 and retesting of samples from the 2008 and 2012 Olympic Games

**DOI:** 10.1186/s40798-020-00293-4

**Published:** 2021-01-07

**Authors:** Alexander Kolliari-Turner, Brian Oliver, Giscard Lima, John P. Mills, Guan Wang, Yannis Pitsiladis, Fergus M. Guppy

**Affiliations:** 1 Collaborating Centre of Sports Medicine, University of Brighton, Eastbourne, UK; 2 Weightlifting Reporter for www.insidethegames.biz and Weightlifting Venue Media Manager London 2012 Olympic Games and Glasgow 2014 Commonwealth Games, Brighton, UK; 3 Department of Movement, Human and Health Sciences, University of Rome “Foro Italico,”, Rome, Italy; 4 School of Sport, Rehabilitation and Exercise Sciences, University of Essex, Colchester, UK; 5 School of Pharmacy and Biomolecular Sciences, University of Brighton, Huxley Building, Lewes Road, Brighton, UK; 6 Centre for Stress and Age-related Disease, University of Brighton, Huxley Building, Lewes Road, Brighton, UK

## Abstract

**Background:**

The pervasiveness of doping and findings of anti-doping corruption threaten weightlifting’s position at the 2024 Olympic Games. Analysing the practices of doping in weightlifters could identify patterns in doping that assist in future detection.

**Methods:**

We analysed publicly available data on sanctioned athletes/support personnel from the International Weightlifting Federation between 2008 and 2019 and announced retrospective Anti-Doping Rule Violations (ADRVs) from the 2008 and 2012 Olympic Games.

**Results:**

There were 565 sanctions between 2008 and 2019 of which 82% related to the detection of exogenous Anabolic Androgenic Steroid (AAS) metabolites and markers indicating endogenous AAS usage. The detection of exogenous AAS metabolites, markers of endogenous AAS usage and other substance metabolites varied by IWF Continental Federation (*p* ≤ 0.05) with Europe (74%, 11%, 15%) and Asia (70%, 15%, 15%) showing a higher detection of exogenous AAS compared to Pan America (37%, 30%, 33%) and Africa (50%, 17%, 33%). When looking at the 10 most detected substances, the nations with the highest number of sanctions (range 17–35) all had at least one overrepresented substance that accounted for 38–60% of all detected substances. The targeted re-analysis of samples from the 2008 and 2012 Olympic Games due to the discovery of long-term metabolites for exogenous AAS resulted in 61 weightlifters producing retrospective ADRVs. This includes 34 original medallists (9 gold, 10 silver and 15 bronze), the highest of any sport identified by Olympic Games sample re-testing. The exogenous AAS dehydrochloromethyltestosterone and stanozolol accounted for 83% of detected substances and were present in 95% of these samples.

**Conclusion:**

Based on these findings of regional differences in doping practices, weightlifting would benefit from the targeted testing of certain regions and continuing investment in long-term sample storage as the sensitivity and specificity of detection continues to improve.

## Key points

- The nations with the highest number of sanctioned weightlifters between 2008 and 2019 (the worst period of doping in weightlifting’s history) all had at least one overrepresented detected substance that accounted for 38–60% of all detected substances.
- Improvements in the detection window for exogenous anabolic androgenic steroids resulted in samples from the 2008 and 2012 Olympic Games being re-analysed and 61 weightlifters produced retrospective Anti-Doping Rule Violations with the highest number of medallists (34) across all sports.
- These findings suggest that weightlifting would benefit from the targeted testing of certain regions and further invest in long-term sample storage at other major competitions (i.e. World and Continental Companionships).

## Introduction

By June 2017, a targeted re-analysis of samples collected from the Beijing 2008 and London 2012 Olympic Games, in which a total of 515 weightlifters competed, had resulted in thirty weightlifters having their medals rescinded as they had retrospectively been identified to have committed an anti-doping rule violation (ADRV) [1, 2]. At this time Thomas Bach, the International Olympic Committee (IOC) President, said weightlifting had ‘a massive doping problem’ [3] and the IOC Executive Board instructed the International Weightlifting Federation (IWF) to demonstrate by December 2017 that it had addressed, or had put in place plans to address, the serious incidence of doping if the sport was to be considered for inclusion in the 2024 Olympic Games [4]. This targeted reanalysis took advantage of improvements in the detection window of exogenous Anabolic Androgenic Steroids (AAS) via the discovery of long-term metabolites (LTMs) [5] for compounds such as Metandienone [6], Dehydrochloromethyltestosterone [7] and Stanozolol [8].

In response, the IWF created two new independent commissions to advise on anti-doping policy changes which respectively became the Clean Sport and Sport Programme Commissions [9, 10]. Additionally, the IWF started a series of actions to combat doping and in 2017 announced 1-year suspensions for nine Member Federations (MFs) found to have had three or more ADRVs from the retesting of samples taken at the 2008 and 2012 Olympic Games [11]. The IWF also enforced a new qualification system for the Tokyo 2020 Olympic Games [12], and each athlete must compete in a minimum of six eligible events that occur within defined time frames to increase the likelihood of being tested in-competition prior to the Olympic Games. This will include at least one event between October 1, 2020, and April 30, 2021, to account for the coronavirus pandemic delaying the Olympic Games [12, 13]. The IWF also announced limitations on MFs for participants per country for the 2020 Olympic Games based on the MFs doping record since the start date of the 2008 Olympic Games and the end of the 2020 qualification period [12]. MFs that had 20 or more ADRVs would be able to send only one male and one female athlete in total; MFs that recorded 10–19 ADRVs would be eligible to send two male and two female athletes; and MFs with less than ten ADRVs would be eligible to send four male and four female athletes [12]. The IWF also signed an agreement with the International Testing Agency (ITA) to take responsibility for key areas of its anti-doping programme, and once this partnership was finalised the IOC lifted the conditional status of weightlifting for the 2024 Olympic Games, citing the positive steps taken by the IWF to combat doping [14, 15]. However, the IOC still reserves its right to review weightlifting’s place on the 2024 Olympic Games Programme, due to the recent revelations of anti-doping corruption in the sport [16].

The Hungarian Anti-Doping Group (HUNADO), who carried out a large proportion of the anti-doping tests requested by the IWF in the last decade, and both the IWF and ex-President Tamás Aján, who’s tenure started in 2000, have had recent accusations of anti-doping corruption with irregularities in Out of Competition (OOC) testing, urine sample manipulation and the disappearance of positive doping results [17] which eventually resulted in Aján’s resignation in April 2020 [18]. An independent report concluded that HUNADO had acted in accordance with World Anti-Doping Agency (WADA) standards [19]. However, the report concluded that former President Tamás Aján had breached confidential information for the planned dates of OOC testing potentially leaking this information to certain nations or athletes [19]. The IWF also deliberately delayed notifying 18 Azerbaijani athletes of their ADRVs, thus enabling them to win medals at international competitions in 2013 [19]. The report also identified that 21 Turkish weightlifters provided samples resulting in Adverse Analytical Findings (AAFs) during OOC tests, but they were not followed through appropriately as although the IWF president was notified of these AAFs the athletes continued competing and winning medals [19]. These cases, plus 41 hidden cases and 10 possible other cases where the AAFs have not been followed through have been forwarded onto WADA for further investigation [19]. The investigative team also found evidence that an additional 130 samples had been taken but not processed [20]. This was information absent from the original report due to insufficient time to investigate prior to the report deadline [20]. Due to the WADA and the ITA investigation currently being open on this case, it is not publicly known how many ADRVs these unprocessed samples relate to, with WADA ‘monitoring this closely to ensure no case is left unprocessed’ [20].

The aim of this analysis of the doping practices of international weightlifters is to aid the sport fight doping as its ongoing commitment to clean sport is required to allow the sport to be on the 2024 Olympic Games Programme [16]. Even though WADA and ITA investigations are still open in regards to identifying the full extent in which AAFs have been hidden by the ex-IWF president, an analysis of salient prohibited substances noted in sanction data by geographical location can still build a clearer picture of doping practices. This will aid governing bodies and anti-doping authorities in identifying regions with higher rates of doping for improved targeted testing and educational programmes. Additionally, investigating the prevalence of retrospectively identified doping from the re-analysis of samples collected from the Olympic Games will also show if the practice of long-term sample storage has been successful in catching doping weightlifters.

## Methods

### Data entry

Data from 2008 to 2019 were obtained from the IWF Sanction List publicly available on the IWF website [21] in February 2020. For weightlifters who had announced retrospective ADRVs from AAFs noted in the re-testing of samples from either the 2008 or 2012 Olympic Games, data were obtained from the IWF Sanction List [21] and other publicly available web pages: IOC ‘Fight Against Doping’ Press Releases [22], IOC [23, 24] and IWF Event Results Pages [25, 26] and IWF Anti-Doping News Archives [27] in mid-May 2020. All detected substance names were made uniform and identified to the parent compound which generated the noted metabolite.

For classification of substances as a marker indicating endogenous AAS (EAAS) usage, WADA technical documents were utilised [28]. These state that EAAS administration can cause alterations in the markers of the urinary steroid profile which is comprised of androsterone, etiocholanolone, 5α-androstane-3α,17β-diol (5αAdiol), 5β-androstane-3α,17β-diol (5βAdiol), testosterone and epitestosterone. Additionally, the administration of testosterone or its precursors, androstenediol, androstenedione, dehydroepiandrosterone or a testosterone metabolite, dihydrotestosterone or a masking agent such as epitestosterone are proven to alter one or more of the parameters of the urinary steroid profile [29], and therefore, any mention of a component of the urinary steroid profile or these substances was denoted as a marker of EAAS usage.

Each sanction was classified based on (1) the IWF Continental Federation (Africa, Asia, Europe, Oceania, Pan America) and (2) the category of the detected substance/prohibited method as described by the 2019 WADA Prohibited List [30]. Three sanctions were omitted from any analyses that involved comparisons of, or counts of, detected substances/prohibited methods because this information was absent or only the article number that was violated by the Anti-Doping Policy of the IWF was stated.

### Statistical analysis

Fisher’s exact test was used to investigate if four IWF Continental Federations (Europe, Africa, Asia, Pan America) had differences in the detection of exogenous AAS metabolites, markers indicating EAAS usage and other substance metabolites, in a 4 × 3 matrix, from the sanctions between 2008 and 2019 obtained from the IWF Sanction List publicly available on the IWF website [21] in February 2020. An adjusted alpha level of 0.05 was used with the Benjamini–Hochberg [31] false discovery rate method for multiple comparisons. Data analysis was conducted using R version 3.6.3 [32] using the tidyverse [33], data.table [34], rcompanion [35], choroplethr [36] and choroplethrMaps [37] packages. The data files and R code used in this study have been made publicly available online [38].

## Results

### The most frequently detected substances

**Fig. 1:**
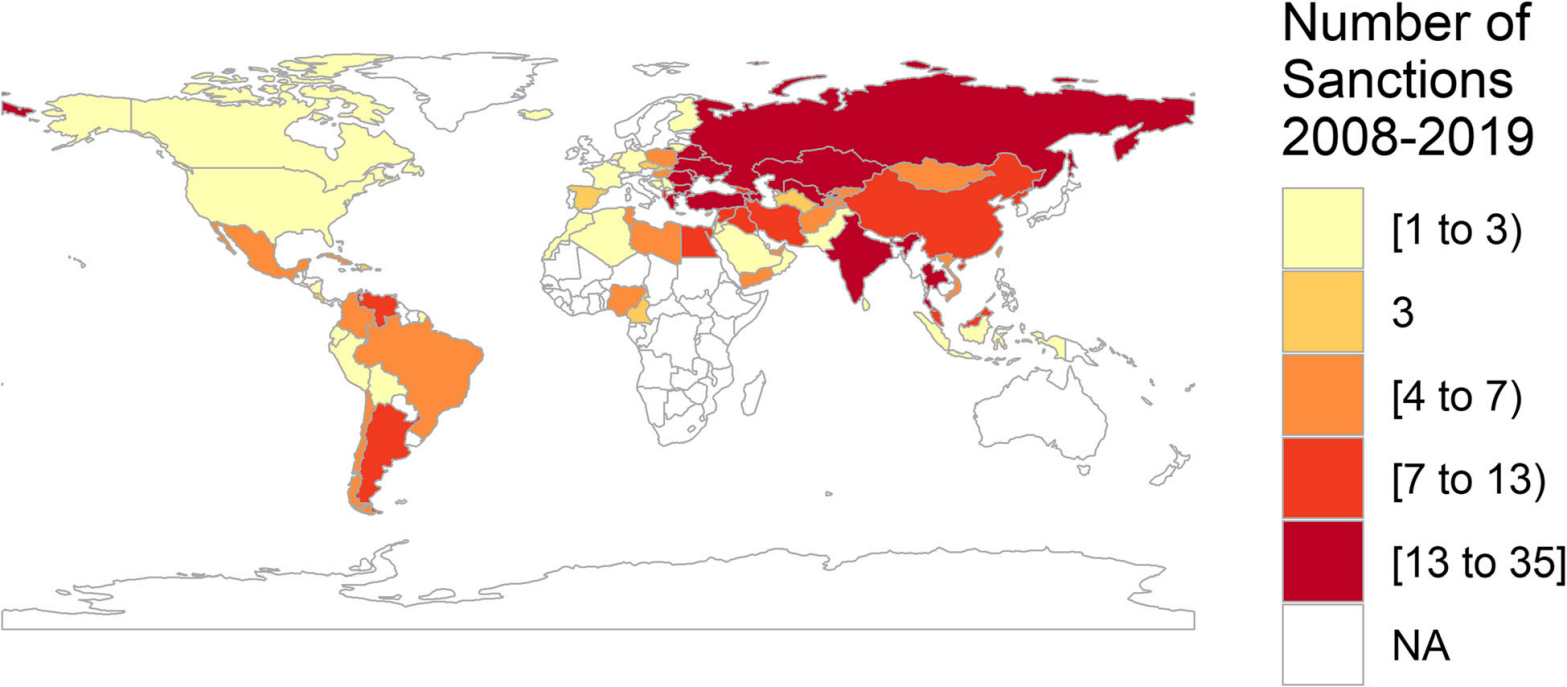
The number of sanctions recorded from the IWF Sanction List [21] between 2008 and 2019 when it was accessed in February 2020 and their geographical location. NA indicates zero-recorded sanctions. Five hundred sixty-five sanctions were recorded but 553 were used for the creation of this figure as the following Member Federations (MF) were not present in the country.map dataset in the choroplethrMaps [36] package in R: Puerto Rico (*n* = 3), Mauritius (*n* = 2), Palestine (*n* = 2), Seychelles (*n* = 2), Aruba (*n* = 1), Barbados (*n* = 1) and Bahrain (*n* = 1)

**Fig. 2:**
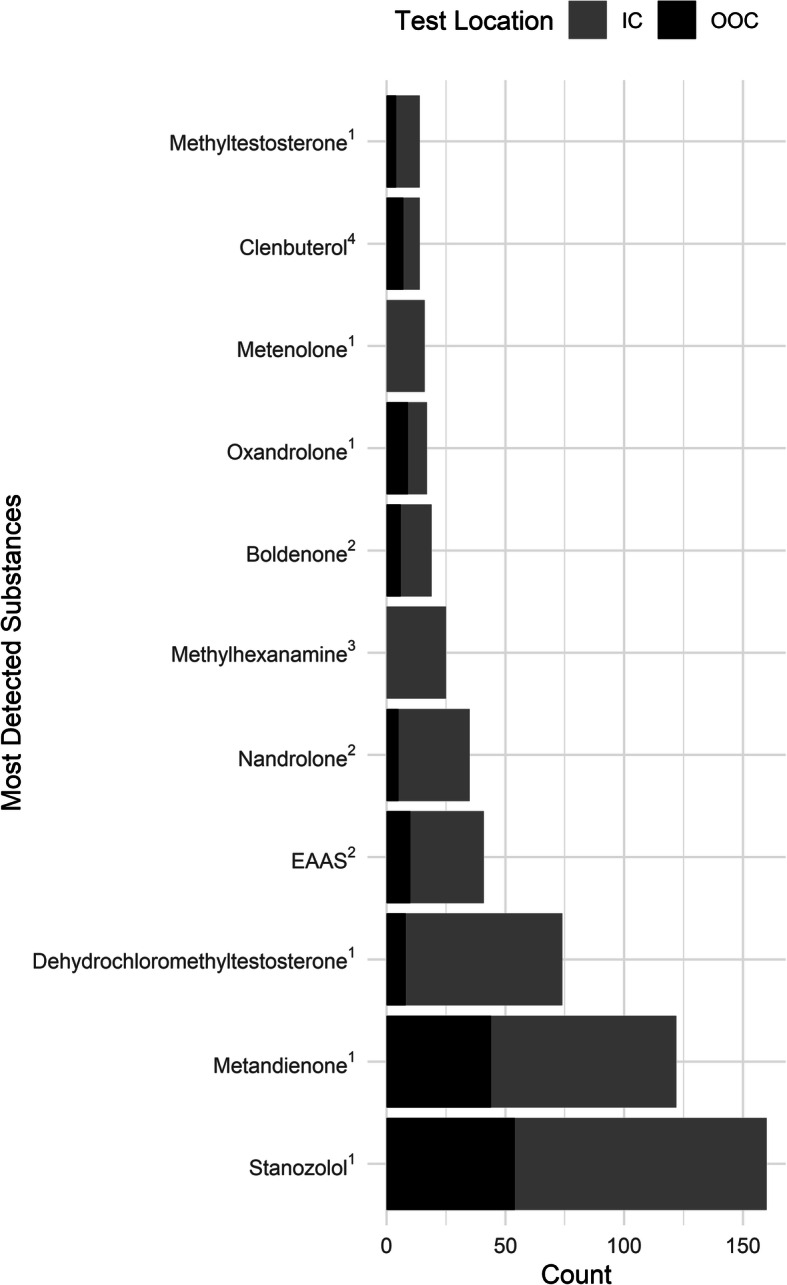
The 10 most detected substances from the IWF Sanction List [21] between 2008 and 2019 and if their detection occurred in-competition (IC) or out-of-competition (OOC) with superscript numbers classifying substances based on the WADA 2019 Prohibited List: exogenous Anabolic Androgenic Steroid (AAS)^1^, markers indicating endogenous AAS usage (EAAS)^2^, Specified Stimulants^3^ and Other Anabolic Agents^4^ [30]. Clenbuterol and Methyltestosterone are tied in 10th place with 14 occasions of detection each. One data point for Methandienone was omitted as the testing location was not defined

Five hundred sixty-five Sanctioned Athletes/Athlete Support Personnel, across 83 different MF, were recorded between 2008 and 2019 (Fig. 1). Five hundred sixty-two of these sanctions had a named prohibited substance/prohibited method noted. Five hundred fifty-nine of these sanctions occurred due to the detection of prohibited substances, with only three sanctions occurring due to the use of prohibited methods (*n* = 2 urine substitution, *n* = 1 blood substitution). Of these 559 sanctions, 51 different substances were detected, from 10 different categories within the WADA Prohibited List, with exogenous AAS metabolites and markers indicating EAAS usage accounting for 82% of detected substances (Fig. 2).

Three hundred ninety-six sanctions occurred from an in-competition (IC) test and 167 from an OOC test with two sanctions testing location undefined. From the ten most detected substances, six substances, Dehydrochloromethyltestosterone (89%), markers indicating EAAS usage (76%), Metenolone (100%), Methylhexanamine (100%), Methyltestosterone (71%) and Nandrolone (86%) showed a higher instance of detection IC (Fig. 2).

### Prohibited substance usage and Continental Federation

Of the 565 Sanctioned Athletes/Athlete Support Personnel counted 199 were from Asia, 267 from Europe, 34 from Africa and 65 from Pan America. There were no sanctions from Oceania. From the 562 sanctions that had the available data, the proportion of detected substances that were classified as exogenous AAS, markers of EAAS usage (i.e. the most detected substances) and all other substance category types varied by IWF Continental Federation (*p* < 0.001). The proportions of these detected substance types was significantly different between Asia (70%, 15%, 15%) and Pan America (37%, 30%, 33%) (*p* < 0.001), Asia and Africa (50%, 17%, 33%) (*p* = 0.039), Europe (74%, 11%, 15%), Pan America (*p* < 0.001), and Europe and Africa (*p* = 0.015) with no differences between Asia and Europe or Pan America and Africa, highlighting regional differences in detected prohibited substances.

### Prohibited substance usage and nation

**Fig. 3:**
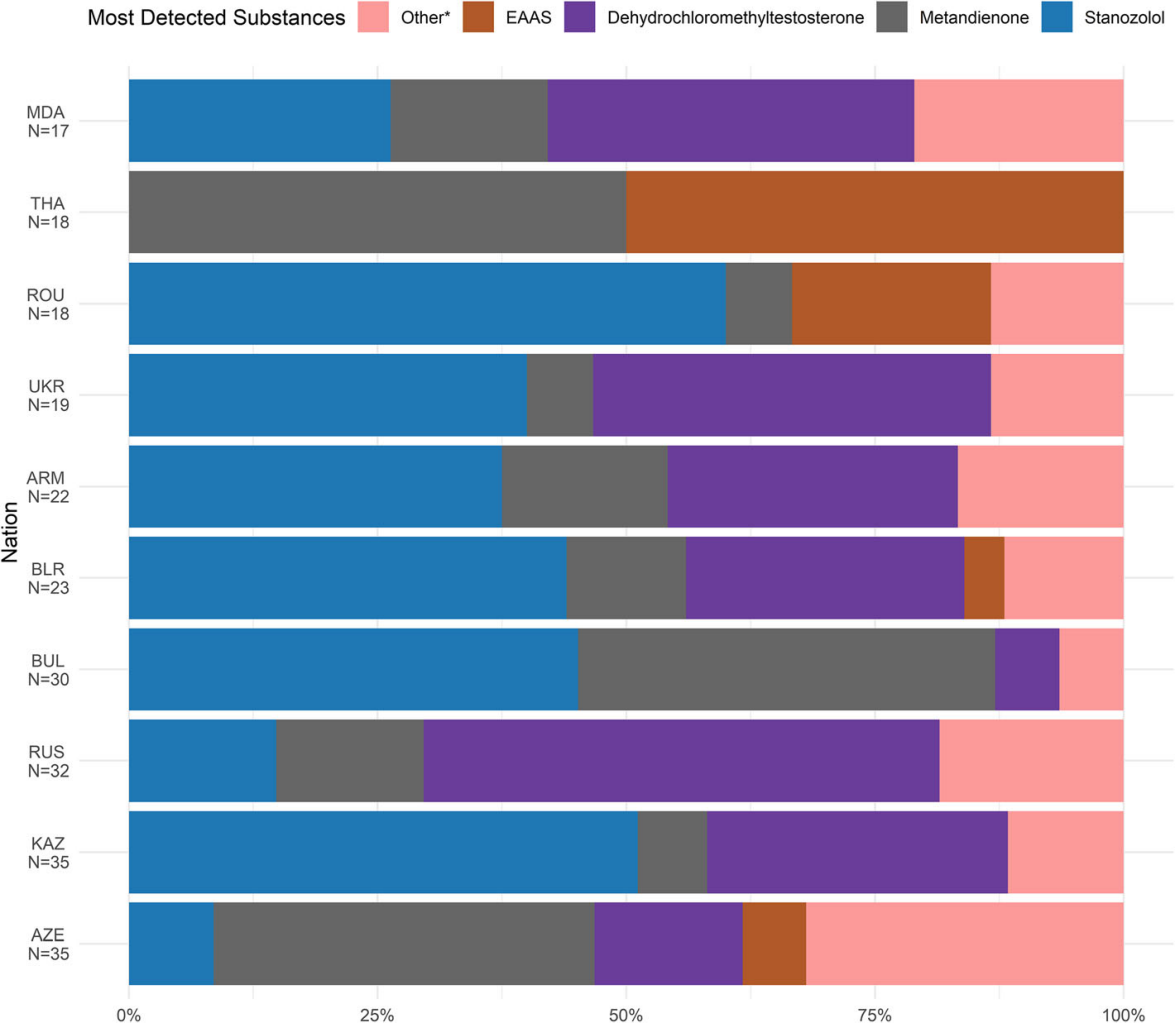
The 10 nations with the highest number of sanctions, from the IWF Sanction List [21] between 2008 and 2019 and for the 10 most detected substances the percentage of times they were detected. Other* denotes either Methyltestosterone, Clenbuterol, Metenolone, Oxandrolone, Boldenone, Methylhexanamine or Nandrolone. EAAS, markers indicating endogenous AAS usage. AZE Azerbaijan, KAZ Kazakhstan, RUS Russia, BUL Bulgaria, BLR Belarus, ARM Armenia, UKR Ukraine, ROU Romania, THA Thailand, MDA Moldova

For the 10 nations with the highest number of sanctions, when looking at the 10 most detected substances, each nation had at least one substance that accounted for more than one third of all detected substances as follows: Azerbaijan (*n* = 35 sanctions) (Metandienone 38%), Kazakhstan (*n* = 35) (Stanozolol 51%), Russia (*n* = 32) (Dehydrochloromethyltestosterone 52%), Bulgaria (*n* = 30) (Metandienone 42% and Stanozolol 45%), Belarus (*n* = 23) (Stanozolol 44%), Armenia (*n* = 22) (Stanozolol 38%), Ukraine (*n* = 19) (Dehydrochloromethyltestosterone 40% and Stanozolol 40%), Romania (*n* = 18) (Stanozolol 60%), Thailand (*n* = 18) (Metandienone 50% and EAAS 50%) and Moldova (*n* = 17) (Dehydrochloromethyltestosterone 37%) (Fig. 3).

### Most affected nations of 2008 and 2012 retesting

**Fig. 4:**
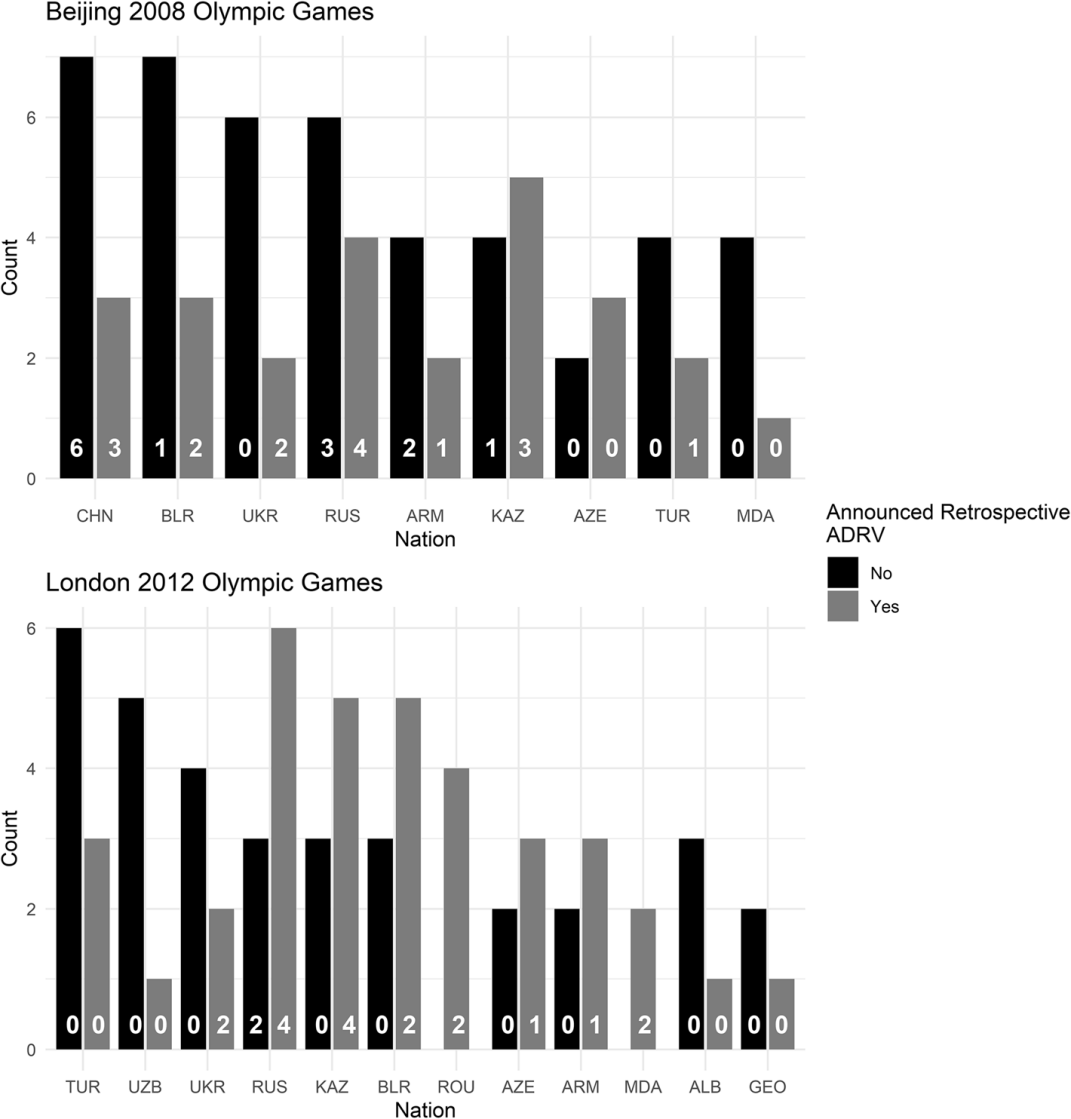
The number of weightlifters that competed from each nation announced to have given retrospective Anti-Doping Rule Violations (ADRVs) via Adverse Analytical Findings (AAFs) from the re-testing of samples collected at the Beijing 2008 and London 2012 Olympic Games. Numbers inside the bars show the number of original medallists. Weightlifters with announced retrospective ADRVs who did not start are included. In Beijing 2008, one athlete from UKR produced an AAF from an in-competition sample and is excluded in these counts. CHN China, BLR Belarus, UKR Ukraine, RUS Russia, ARM Armenia, KAZ Kazakhstan, AZE Azerbaijan, TUR Turkey, MDA Moldova, UZB Uzbekistan, UKR Ukraine, ROU Romania, ALB Albania, GEO Georgia

Sixty-one weightlifters, from 13 different countries, were retrospectively announced to have committed an ADRV for prohibited substances from the Beijing 2008 (*n* = 25) and London 2012 (*n* = 36) Olympic Games. Sixteen of these weightlifters (64%) from Beijing 2008 were medallists (4 Gold, 5 Silver and 7 Bronze). For Beijing 2008 Kazakhstan and Azerbaijan had more athletes generate a retrospective ADRV than those who did not and for Belarus, Ukraine, Russia and Kazakhstan more medals were won by athletes who generated a retrospective ADRV than those who have not (Fig. 4). Eighteen of these weightlifters (50%) from London 2012 were medallists (5 gold, 5 silver and 8 bronze). For London 2012 Russia, Kazakhstan, Belarus, Azerbaijan and Armenia had more athletes generate a retrospective ADRV than those who have not and for both Romania and Moldova all athletes that competed generated a retrospective ADRV. All medallists from Ukraine, Kazakhstan, Belarus, Romania, Azerbaijan, Armenia and Moldova generated retrospective ADRVs and for Russia twice as many medals were won by athletes who generated a retrospective ADRV (Fig. 4).

### Most affected categories of 2008 and 2012 retesting

From Beijing 2008, five weight categories (men’s u94kg, women’s u48kg, women’s u69kg, women’s u75kg and women’s 75 kg+) and from London 2012, three weight categories, (women’s u53kg, women’s u63kg and women’s u69kg) had two medallists produce retrospective ADRVs from AAFs. In two instances from London 2012 (men’s u94kg and women’s u75kg), all medal winners produced retrospective ADRVs from AAFs, with the men’s u94kg category being the worst affected with eight athletes generating retrospective ADRVs from AAFs, six of whom originally placed in the top 10.

### Detected substances from 2008 and 2012 retesting

**Fig. 5:**
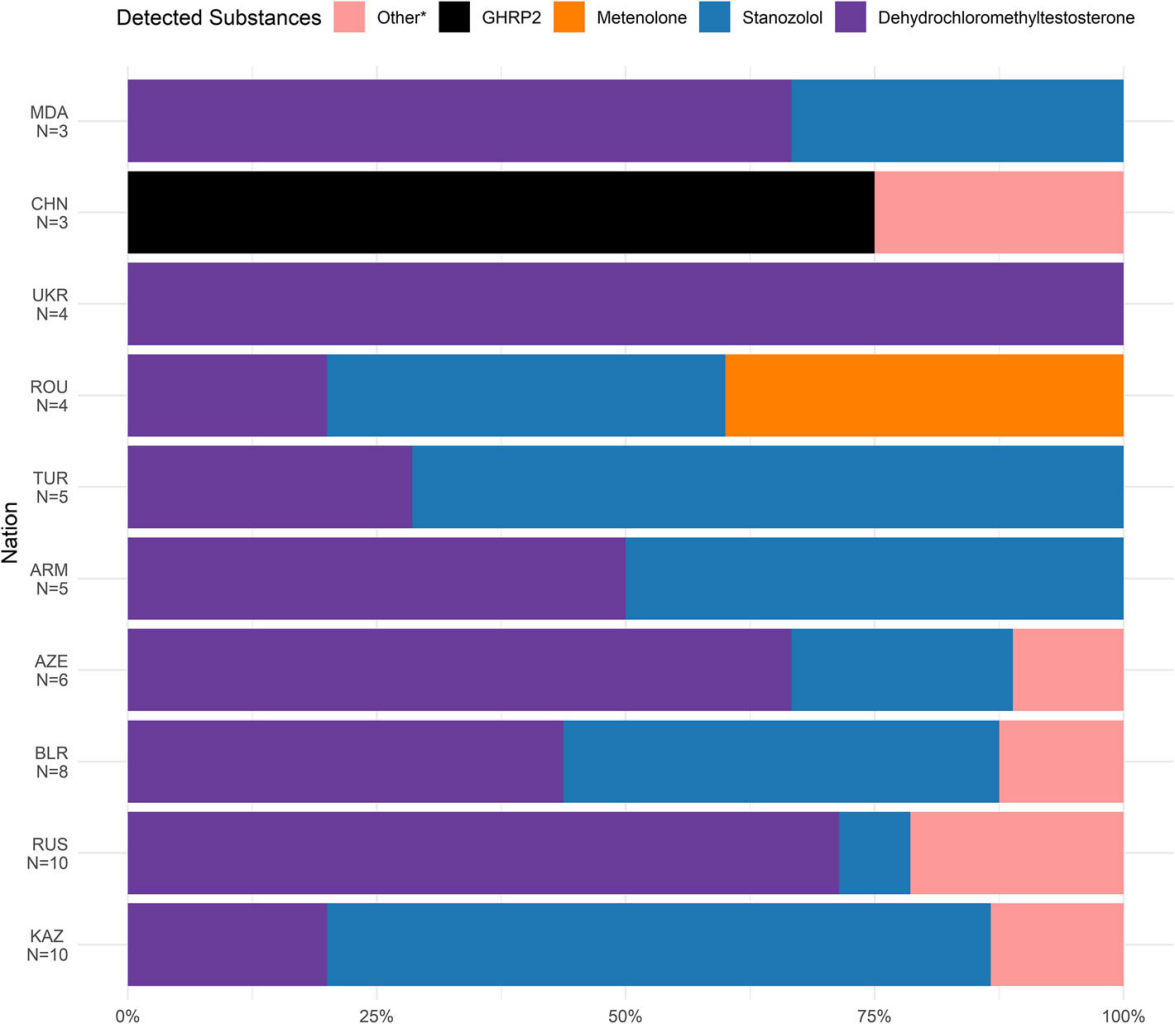
The 10 nations with the highest number of announced retrospective Anti-Doping Rule Violations via Adverse Analytical Findings from both the Beijing 2008 and London 2012 Olympic Games and the percentages of detected substances identified. Other* denotes either Drostanolone, Erythropoietin, Oxandrolone, Sibutramine or Tamoxifen. GHRP2 Growth Hormone-Releasing Peptide-2, KAZ Kazakhstan, RUS Russia, BLR Belarus, AZE Azerbaijan, ARM Armenia, TUR Turkey, ROU Romania, UKR Ukraine, CHN China, MDA Moldova

In total, across both the Beijing 2008 and London 2012 Olympic Games, 94 prohibited substances were detected in the re-tested samples with Dehydrochloromethyltestosterone and Stanozolol accounting for 83% of all detected substances. The majority of retrospective ADRVs (58 of 61) were caused by the detection of one of these two substances with exogenous AAS accounting for 94% of all detected substances. Across both Olympic Games, for the 10 nations with the highest number of announced retrospective ADRVs, the proportions of detected substances are shown in Fig. 5. For each nation there is at least one substance that makes up ≥ 40% of all detected substances as follows: Kazakhstan (*n* = 10 ADRVs) (Stanozolol 67%), Russia (*n* = 10) (Dehydrochloromethyltestosterone 71%), Belarus (*n* = 8) (Dehydrochloromethyltestosterone 44%, Stanozolol 44%), Azerbaijan (*n* = 6) (Dehydrochloromethyltestosterone 67%), Armenia (*n* = 5) (Dehydrochloromethyltestosterone 50%, Stanozolol 50%), Turkey (*n* = 5) (Stanozolol 71%), Romania (*n* = 4) (Metenolone 40%, Stanozolol 40%), Ukraine (*n* = 4) (Dehydrochloromethyltestosterone 100%), China (*n* = 3) (Growth Hormone-Releasing Peptide-2 75%) and Moldova (*n* = 3) (Dehydrochloromethyltestosterone 67%).

## Discussion

The time period of this analysis has seen the highest number of sanctions in weightlifting’s history. It has also seen an independent investigation into allegations of anti-doping corruption finding the IWF president to have breached the confidentiality of the planned timing of OOC sample collection, potentially giving advanced notice of OOC testing to individual countries or athletes [19]. The president also delayed the announcement of ADRVs from 18 Azerbaijani weightlifters and AAFs from 21 Turkish weightlifters were not followed through appropriately, enabling them to win medals in international events. Investigations by WADA and the ITA are still pending on an additional ‘41 hidden cases and 10 possible other cases where the AAFs have not been followed through’ [19] and on 130 unprocessed samples (in which the number of AAFs is unknown) [20]. Despite these ongoing investigations, this study intended to build a clearer picture of doping practices of weightlifters and how these practices varied across the IWF Continental Federations, based on known sanction data, to enhance future doping detection and to investigate the re-testing of samples collected at the 2008 and 2012 Olympic Games.

Over an 11-year period, exogenous AAS metabolites and markers indicating EAAS usage accounted for 82% of detected substances from the IWF Sanction List. The effects of AAS on increasing skeletal muscle mass and strength have been well documented [39–44], and these ergogenic benefits are a likely reason for their preference of usage by doping athletes who compete in a strength sport. Europe generated the highest number of sanctions (*n* = 267) followed by Asia (*n* = 199), Pan America (*n* = 65) and Africa (*n* = 34), with no sanctions from Oceania recorded. During this time frame, weightlifting has been most popular in Europe and Asia, but globally popularity has been expanding with the senior 2019 Oceania [45], African [46] and Pan American [47] Championships hosting 129, 112 and 187 weightlifters, respectively, whilst the senior 2019 European [48] and Asian [49] Championships hosted 322 and 214 weightlifters, respectively.

The proportions of detected exogenous AAS metabolites, markers indicating EAAS usage and all other substance category types varied by IWF Continental Federation. Europe and Asia both respectively showed statistically different (*p* < 0.05) proportions of detection for these three substance types compared to both Africa and Pan America with exogenous AAS showing the largest difference in the proportion of substance types detected. The most detected exogenous AAS were Stanozolol, Metandienone and Dehydrochloromethyltestosterone. At the national level there is also differences in the detection of substances as when looking at the 10 most detected substances, from the 10 nations with the highest numbers of sanctions, there is at least one substance overrepresented that accounts for 38–60% of detected substances in these countries (Fig. 3). The cultural preference of certain doping substances at the regional/national level is likely heavily confounded by regional/national drug availability (legally or illicitly). Nationally, the continued usage of certain substances could be decentralized (e.g. athletes’ independent choice based on availability) or centralized (i.e. state-sponsored doping). Making inferences on the cause of substance over-representation from substance detection data is not possible, but identifying these patterns is useful knowledge for anti-doping authorities as they could use this data for targeted educational programmes to illicit the change required to change these patterns. These patterns corroborate with the notion from the independent report into Anti-doping corruption into weightlifting that although the ex-president ‘interfered with the IWF Anti-Doping Commission, *the real problem is the culture of doping that exists in the sport*.’ [19]. Additionally, these geographic differences in doping practices could better inform targeted testing applied by anti-doping authorities and targeted investigations into other ways of identifying ADRVs such as trafficking, aiding/abetting and complicity. For effective doping control, international sporting authorities should have anti-doping programmes that frequently conduct unannounced OOC testing, across all regions of the globe, to catch doping athletes who intend for prohibited metabolites to clear their urine prior to anticipated IC tests. With Europe and Asia showing the highest number of sanctions and highest prevalence in the usage of exogenous AAS an extra emphasis on OOC testing in these regions may be warranted in weightlifting as these substances are likely to be used in training prior to competition where anticipated testing occurs. Additionally, if athletes from these regions now consider that the likelihood of getting caught using exogenous AAS is now high due to their high detection prevalence they may now start to use other performance enhancing drugs (PEDs) with shorter detection windows meaning that OOC testing is even more important.

The decision of the IOC to store athletes’ samples collected from Olympic Games for 10 years has proven particularly fruitful for catching doping medallists in weightlifting. This analysis of re-tested samples has shown that the intention of doping athletes who ceased the usage of exogenous AAS prior to the 2008 and 2012 Olympic Games with the aim of diagnostic metabolites clearing their urine prior to an anticipated test was successful (with the known metabolites and detection science at the time of these Olympic Games). However, once the LMTs for exogenous AAS were discovered via improvements in highly sensitive detection methods employing chromatographic/mass spectrometric techniques [5], a doubling of the detection window [6, 8] occurred for some exogenous AAS and subsequently 34 medallists were caught doping retrospectively when these samples were reanalysed. From the sixty-one retrospective ADRVs identified via re-testing, the exogenous AAS Dehydrochloromethyltestosterone and Stanozolol accounted for 83% of detected substances with 95% of announced athlete ADRVs noting at least one of these substances. These findings should send a strong deterrent to prospective doping athletes that, due to LMT discovery, the detection window of these substances has substantially improved and the doping practices of athletes in the runup to the 2008 and 2012 Games may not be possible anymore for future competitions. The IOC has announced that the ITA has planned the ‘most comprehensive pre-Games testing programme ever conducted’ for Tokyo 2020 and that $5 million, spread over 10 years, will be allocated to a comprehensive long-term storage programme [50, 51], potentially acting as a stronger deterrent to prospective doping weightlifters. However, a long-term storage is not standard across Continental Games, with the International Federations (IF) having to fund the cost of a long-term storage [52]. Based on the success shown with weightlifting, the IWF and other IF should further their investment in a long-term sample storage at Continental Games and other important international competitions. Other categories of PEDs that may currently have shorter detection windows may be used instead of exogenous AAS by weightlifters, or other athletes in strength/power sports (e.g. EAAS), due to this improved detectability in exogenous AAS, but these shorter detection windows could improve in the future (e.g. by advances in the steroidal module of the athlete biological passport), and thus, a long-term sample storage would enable a re-analysis of samples with improved detectability.

## Conclusion

This analysis of doping practices, over a period of 11 years, has shown avenues that may enhance the future detection of doping weightlifters. For example, with Europe and Asia producing the highest numbers of sanctioned weightlifters, as well as the highest prevalence in the detection of exogenous AAS, higher rates of targeted OOC testing in these regions may be warranted, both in the instance that these substances continued to be used or if a transition is made to substances with shorter detection windows. Educational programmes on anti-doping may also be required to change the behaviour in nations with the highest number of sanctions especially focussing on detected substances that are overrepresented in their doping weightlifters. Lastly, the prevalence of retrospectively identified doping at the Beijing and London Olympic Games shows that the long-term storage of samples should continue, with the aim of increasing this practice at additional competitions to the Olympic Games, as anti-doping science continues to improve its detection methods.

## Data Availability

The CSV files that contain the data from this study and R Code used in this study are publicly available on OSF: https://osf.io/8j6ya/.

## Abbreviations

AAF: Adverse Analytical Findings
AAS: Anabolic Androgenic Steroids
ADRV: Anti-Doping Rule Violations
EAAS: Endogenous Anabolic Androgenic Steroids
HUNADO: Hungarian Anti-Doping Group
IC: In-competition
IOC: International Olympic Committee
ITA: International Testing Agency
IWF: International Weightlifting Federation
LTMs: Long-term metabolites
MF: Member Federations
OOC: Out of competition
PED: Performance Enhancing Drug
WADA: World Anti-Doping Agency
